# Selections

**Published:** 1893-01

**Authors:** 


### Selections.

### HYDRONAPHTHOL IN THE PROPHYLAXIS AND TREATMENT OF CHOLERA.

DR. D. D. STEWART, of Philadelphia, suggests hydronaphthol as both a preventive and as a remedy for the cholera, after several years' experience with this drug in intestinal affections of bacterial origin. A remedy to be directed with effect against the contagion of cholera should be a more or less ideal antiseptic; it should be but slightly soluble and decomposable, yet a germicide in aqueous solution, and both non-toxic and non-irritant in doses sufficient to produce a germicidal action. It occurred to the author that hydronaphthol would perhaps fulfil the desired indications. It has been found of signal service in intestinal affections, especially those of bacterial origin. It is related to phenol, being, like it, a benzol derivative; hence, presumably, cholera spirilla might also be vulnerable to it. It is but slightly soluble in aqueous solutions, is non-irritant and non-poisonous, and does not readily undergo decomposition; it would, therefore, be carried unchanged to the affected part of the bowel, even in moderate doses, without absorption occurring. To determine its actual value as an antiseptic and germicide in cholera, Dr. Ghriskey, of the Laboratory of Hygiene, University of Pennsylvania, undertook some experiments as to its influence on pure cultures of comma-spirilla. It was found in a series of experiments that in solutions of a strength of one part hydronaphthol and seven thousand parts nutritive culture-medium, the drug proved distinctly antiseptic. It was also demonstrated that with a mixture of equal parts of a saturated aqueous solution of hydronaphthol and a bouillon-culture of this organism, the drug was germicidal within five minutes. Dr. Stewart thinks we are likely to have in it a medicament of extraordinary value, for it has been actually demonstrated beyond question that a proportion as high as one to seven thousand has an undoubted inhibiting effect on the development of the cholera-spirillum, and that a proportion of about one to two thousand exerted a prompt germicidal action. As one part to seven thousand equals about a grain to the pint, or to the avoirdupois pound, and as the contents of the small intestine, when the latter in its entire length is thoroughly distended, cannot amount to more than nine or ten pints, it would follow that, under any condition, but ten grains of hydronaphthol, if in solution, would be required to render the entire small intestine antiseptic against the comma-spirillum, preventing its development, while about forty grains, under similar conditions, would disinfect the intestine, promptly killing any spirilla present. Fortunately hydronaphthol is non-toxic in doses probably much larger than would be sufficient for the latter effect. In cases of simple diarrhœa, in dysentery, and in enteric fever, the author has frequently administered a half-drachm in the twenty-four hours, continuing this often for weeks, totally without effect other than beneficial, but he thinks it certain that doses much larger than these may be similarly used. As a prophylactic against cholera, when, from exposure, the disease seems imminent, hydronaphthol should be taken in doses of from eight to ten grains four times daily for three or four days.—*Medical News*, October 1, 1892.

